# Crossbreeding parameters for body weight data from a complete diallel mating scheme using three breeds of rabbit

**DOI:** 10.5194/aab-67-335-2024

**Published:** 2024-07-05

**Authors:** Ahmed M. Abdel-Ghany, Salah A. El-Mansy, Dalal S. Alshaya, Nora M. Al Aboud, Mahmoud G. Gharib

**Affiliations:** 1 Department of Animal Production, Faculty of Agriculture, Suez Canal University, Ismailia 41522, Egypt; 2 Department of Animal Production, Faculty of Agriculture, Zagazig University, Zagazig 44511, Egypt; 3 Department of Biology, College of Science, Princess Nourah bint Abdulrahman University, P.O. Box 84428, Riyadh 11671, Saudi Arabia; 4 Department of Biology, Faculty of Science, Umm Al-Qura University, Makkah, Saudi Arabia; 5 Animal Production Research Institute (APRI), Agricultural Research Center (ARC), Giza, Egypt

## Abstract

The objective of this study was to evaluate heterosis, general combining ability (GCA), maternal ability ( $G^{M}$ ), and sex-linked effects (SL) for growth performance from weaning (at 4 weeks) up to marketing age (at 12 weeks) using a complete 3×3 diallel crossing experiment in three different breeds of rabbit: one native Egyptian (Baladi Red, RR) and two exotic (Bauscat, BB, and Californian, CC). Offspring (2617 rabbits) body weight data (BW; 4–12 weeks of age) were analyzed, mainly to evaluate the effects of mating groups (MGs), sex, year season, and parity. MGs were further analyzed for crossbreeding parameters. All crossbreds showed positive, highly significant values ( P≤0.001 ). Heterosis ( $H^{I}$ ; overall or specific) for body weight at all evaluated ages was highly significant ( P≤0.001 ). Purebred differences were affected significantly ( P≤0.01 or P≤0.001 ). No significant effects were detected due to general combining ability, maternal abilities, or reciprocal effect (sex-linked). Results suggest that to some extent and because of the highly purebred significant differences ( P≤0.01 or P≤0.001 ), those three breeds are expected to be too genetically far apart to make crosses showing significant heterosis. Since other crossbreeding effects are non-significant, heterosis is the only reliable effect that can produce the Egyptian broiler rabbits when using those three breeds. However, the insignificance of the reciprocal impact (sex-linked as a whole or, by definition, the additive effects of genes carried on the sex chromosomes) of the two acclimatized standard breeds (CC and BB) seemed to be of magnitude at least to the 10th week of age.

## 1 Introduction

Rabbit meat is considered a functional food due to its high nutritional properties; it is lean, easily digestible, and rich in biologically valuable proteins and contains high levels of essential amino acids (Ashour et al., 2014; Abdelnour et al., 2020a, b; Sheiha et al., 2020; Abou-Kassem et al., 2021; Mohamed et al., 2023), and it has low contents of fat, cholesterol, and sodium. At the same time, it has a high content of unsaturated fatty acids (UFA; especially *ω -3 and ω -6) and a good ratio of polyunsaturated fatty acids (PUFA). It is also a very good source of minerals (P, K, Ca, Se, and Co) and vitamins (vitamins B ${}_{2}$ , B ${}_{3}$ , B ${}_{5}$ , B ${}_{6}$ , B ${}_{12}$ , and niacin) (Frunză et al., 2023). Therefore, genetic improvement of rabbits' economic traits is important to increase their contribution to the much-needed animal protein (Akanno and Ibe, 2005).

Body weight, primarily expressing growth, especially early in an animal's life, is notably an important economic trait. This quantitative economic trait is improved by crossing the local Baladi Red breed with Bauscat rabbits (Abdel-Ghany et al., 2007). The improvement of rabbit breed productivity can be achieved by crossbreeding or selection. There are several advantages delivered by crossbreeding and termination of aggregate cumulative interbreeding, which occurs through the continuous selection process (Adenaike et al., 2013).

Diallel crossing is a valuable technique for harnessing the benefits of heterosis between parental populations and their offspring. It is commonly employed in rabbit breeding programs to enhance growth traits (Abdel-Hamid, 2015; Kariman-Farg et al., 2021; Setiaji et al., 2022). The diallel cross is a mating scheme utilized to investigate the genetic underpinnings of quantitative traits. It holds enormous potential for enhancing production performance in animal and poultry breeding programs. Various modifications have been developed to fully leverage the advantages of diallel crossing, including full, partial, incomplete, and complete (Dubey et al., 2020). The combining ability in the cross has been described as the ability of parents to bond with each other during fertilization so that genes or characters can be transmitted to their offspring (Henderson, 1952).

The objective of this study was to estimate heterosis, general combining ability (GCA), maternal ability ( $G^{M}$ ), and sex-linked effects (SL) for growth performance from weaning (at 4 weeks) up to marketing age (at 12 weeks) using a diallel crossing scheme, involving three imported breeds, Baladi Red (RR; an indigenous Egyptian breed) and Bauscat (BB) and Californian (CC)(two exotic breeds), to identify the most optimal cross-combination for enhanced growth performance in broiler rabbit.

## 2 Materials and methods

### 2.1 Source of the experimental diets

Experimental work was conducted at the Rabbit Farm of the Animal Production Department, Faculty of Agriculture, Suez Canal University, Al-Ismailia, Egypt, during 3 consecutive years of production. The studied animals involved one native breed of rabbits (Baladi Red, RR) and two exotic breeds (i.e., Bauscat, BB, and Californian, CC).

All does and bucks of either native or exotic rabbit breeds were raised under the same managerial, environmental, and veterinarian conditions. Mating was arranged in a 3×3 full diallel crossbreeding scheme, with a tendency to increase the numbers of purebreds.

A description of mating groups used to assess those three purebreds and their reciprocal crosses is presented in Table 1. Rabbits were raised in a semi-closed rabbitry. Breeding does and bucks were housed separately in individual wire cages arranged in a single-tier battery. According to the established breeding plan, each doe was introduced to the cage of her rotationally assigned buck for mating. Upon successful mating, the doe was returned to her cage. A period of 10 d later, each doe underwent palpation to determine pregnancy. If a doe failed to conceive, it was reintroduced to the same mating buck until successful mating occurred.

**Table 1:** Number of animals for various mating groups (i.e., progeny, sires, and dams).

Mating groups of rabbits	Breed of sire	Breed of dam	Number of		
			progeny	sires	dams
Purebreds					
Bauscat (B)	B	B	956	25	62
Californian (C)	C	C	929	33	74
Baladi Red (R)	R	R	231	10	27
Crossbred ${}^{*}$					
BC	B	C	90	7	12
BR	B	R	87	6	11
CR	C	R	78	5	10
Reciprocals ${}^{*}$					
CB	C	B	74	7	11
RB	R	B	85	4	11
RC	R	C	87	7	11
Total			2617	104	229

${}^{*}$ Sire breed was introduced first.

Weaning occurred at 28 d after birth. At that time, the young rabbits were sexed, ear-tagged, and transferred to new batteries. They were housed in groups of three to four individuals in standard progeny wire cages equipped with feeding hoppers and drinking nipples. The pregnant, lactating does and young rabbits were fed ad libitum with a commercial pelleted growing ration formulated to provide approximately 18 % crude protein, 13% crude fibers, and 2.5 % fat (digestible energy = 2500 kcal per kg diet). In contrast, bucks and empty does are given 130–150 g daily to prevent over-fattening. In contrast, bucks and empty does are fed 130–150 g daily to avoid over-fattening.

### 2.2 Statistical analysis

A dataset of 2617 offspring was analyzed using the least-squares method to assess the influence of mating groups. Subsequently, crossbreeding parameters were further evaluated for traits considered significant.

Data for growth traits (i.e., weaning and post-weaning body weights, denoted as BW ${}_{i}$ ; i = 4, 6, 8, 10, and 12 weeks of age) were analyzed using the general linear model (GLM) procedure developed by SAS (2004). The following mathematical model was used:

1 $$\begin{array}{rl} Y_{gsflmn} & =\mu +{\mathrm{MG}}_{g}+{\mathrm{SEX}}_{s}+(Y-S{)}_{f}+P_{l}\\ & +\beta (\mathrm{LSB}{)}_{m}+e_{gsflmn}, \end{array}$$

where $Y_{gsflmn}$ is the observation of body weight; μ is an underlying constant, which is the overall least-squares mean specific to each trait; MG ${}_{g}$ is the fixed effect of the g th mating groups; SEX ${}_{s}$ is the fixed effect of the s th sex; $(Y-S{)}_{f}$ is the fixed effect of the f th year season of birth; $P_{l}$ is the fixed effect of l th parity; litter size at birth (LSB) is a covariate; and $e_{gsflmn}$ denotes the random residuals which are assumed to be independent and identically normally distributed.

Diallel crossbreeding effects were estimated (based on Griffing, 1956, and Harvey, 1960) as follows:

2 $$Y_{hijk}=\mu +a_{h}+p_{ii}+g_{i}+g_{j}+m_{j}+r_{ij}+e_{hijk},$$

where $y_{hijk}$ is the k th observation on the progeny of the i th sire breed and j th dam breed (mating group), in the h th type of breeding (purebred or crossbred); μ is the overall least-squares mean; $a_{h}$ is an effect common to all progenies of the h th type of breeding; $p_{ii}$ is the purebred effect common to all progeny of a mating between the i th sire breed and i th dam breed; $g_{i}$ is the general combining ability effect for the i th breed; $g_{j}$ is the general combining ability effect for the j th breed; $m_{j}$ is the maternal ability effect of the j th breed of the dam; $r_{ij}$ is the residual reciprocal effect or sex-linked effect in the progeny of the i th sire breed and j th dam breed; and $e_{hijk}$ is a random error, normally and independently distributed (NID; 0, $\sigma^{2}e$ ). All crossbreeding effects were estimated and evaluated herein through contrasts. All contrasts were tested against the main ANOVA's experimental (residual) error. Those combined contrasts (i.e., GCA; $G^{M}$ ; …) were tested similarly.

The heterotic effects (overall, $H^{O}$ ) were calculated using the following formulae.

$$\begin{array}{c} \begin{array}{rl} & \mathrm{heterosis}\;(\mathrm{overall},\;\mathrm{linear}\;\mathrm{function},\;H^{O})\\ & \;=\{[\mathrm{all}\;\mathrm{crosses}\;\mathrm{and}\;\mathrm{reciprocals}]-[\mathrm{all}\;\mathrm{purebreds}]\}. \end{array}\\ \begin{array}{rl} & \mathrm{heterosis}\;(\text{linear function for a specific cross},\;H^{I})\\ & \;=[F_{1}-\{(P_{1}+P_{2})/2\}]/\{(P_{1}+P_{2})/2\}, \end{array} \end{array}$$

where $F_{1}$ is the mean of the two reciprocal crossbreds, and $P_{1}$ and $P_{2}$ are the means of parental breeds used to produce this crossbred.

This model was employed to evaluate the statistical significance and estimate the impact of overall and specific-cross ( $H^{I}$ ) heterosis, purebreds, maternal ability ( $G^{M}$ ), general combining ability (GCA), and sex-linked effects, applying the restrictions by Harvey (1960).

The restrictions for that crossbreeding model are as follows:

3 $$\begin{array}{rl} \sum_{h}a_{h} & =\sum_{i}p_{ii}=\sum_{i}g_{i}=\sum_{j}g_{j}=\sum_{j}m_{j}=\sum_{i}r_{ij}\\ & =\sum_{j}r_{ji}=r_{ij}+r_{ji}=0. \end{array}$$

## 3 Results and discussion

### 3.1 Non-genetic effects

The present results in Table 2 showed that sex differences in body weight between male and female rabbits were significant ( P≤0.001 ) at all evaluated ages except at weaning, where male rabbits were always heavier than females. It was observed that year season combinations of birth and parity effects on body weights of growing rabbit's frameworks were highly significant ( P≤0.001 ) at all evaluated weeks of age. Analogously, the results of Lazzaroni et al. (2012) and Apori et al. (2015) ascertained a significant year of birth effect ( P≤0.01 or P≤0.001 ) on body weight at different ages. Kariman-Farg et al. (2021) noted that, at various studied ages, there was an insignificant effect of sex on the body weight of growing rabbits.

**Table 2:** F ratios of least-squares analysis of variance for different factors affecting rabbits' body weight traits at different evaluated ages. DOF: degrees of freedom.

Sources of variation	DOF	BW ${}_{4}$ ${}^{a}$	BW ${}_{6}$	BW ${}_{8}$	BW ${}_{10}$	BW ${}_{12}$
Mating groups (MGs)	8	8.71 ${}^{***}$	12.63 ${}^{***}$	6.25 ${}^{***}$	4.99 ${}^{***}$	5.46 ${}^{***}$
SEX	1	1.86	11.28 ${}^{***}$	22.38 ${}^{***}$	37.08 ${}^{***}$	52.82 ${}^{***}$
Birth year season	11	7.79 ${}^{***}$	8.20 ${}^{***}$	6.68 ${}^{***}$	5.50 ${}^{***}$	5.02 ${}^{***}$
Parity	4	9.24 ${}^{***}$	7.81 ${}^{***}$	6.97 ${}^{***}$	5.96 ${}^{***}$	4.47 ${}^{***}$
LSB (covariate)	1	27.84 ${}^{***}$	46.94 ${}^{***}$	35.89 ${}^{***}$	25.61 ${}^{***}$	19.96 ${}^{***}$
Error DOF		2566	2358	2208	2122	2132

${}^{a}$ BW ${}_{4}$ , BW ${}_{6}$ , BW ${}_{8}$ , BW ${}_{10}$ , and BW ${}_{12}$ are body weight (g) at 4, 6, 8, 10, and 12 weeks, respectively. BB is Bauscat, CC is Californian, and RR is Baladi Red. ${}^{***}$ Significant ( p≤0.001 ).

### 3.2 Mating groups (MGs)

Data in Table 2 show that the breed groups and different factors showed high significance ( P≤0.001 ) for BW during all interval ages. In this respect, Maj et al. (2009), El-Bayomi et al. (2012), Adenaike et al. (2013), Kabir et al. (2014), Abdel-Hamid (2015), and Kariman-Farg et al. (2021) reported that different breeds of rabbits significantly influence on body weight at different ages. Data presented in Table 3 showed the least-squares means (mean ± standard error) for BW traits at different ages for the nine genotypes produced from the “ 3×3 ” full diallel cross. Results revealed that the CC rabbits exhibited the highest BW figures, followed by the BB rabbits, compared to RR rabbits at most evaluated ages. However, as regards the crosses' BWs, the superiority was insignificantly ( P>0.05 ) exchanged between the two crosses, BC (breed combination; Bauscat 
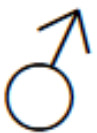
 × Californian 
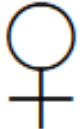
) and CR (cross; Californian 
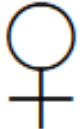
 × Baladi Red 
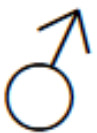
); the latter achieved the highest apparent superiority in the second half of the evaluated growing period (10–12 weeks of age) and the second highest in most of the early growth stages. The present findings were similar to those found by El-Bayomi et al. (2012) on New Zealand white, Californian, and Gray Giant Flander breeds; Abdel-Hamid (2015) on New Zealand white, Californian, and Rex breeds; and Kariman-Farg et al. (2021) on New Zealand white, Papillon, and Flemish Giant breeds.

**Table 3:** Least-squares mean (LSM) ± standard error (SE) rabbits' post-weaning body weight traits at different evaluated ages (4–12 weeks of age) for all purebred and crossbred rabbits.

Mating groups	LSM ± SE				
	BW ${}_{4}$ ${}^{b}$	BW ${}_{6}$	BW ${}_{8}$	BW ${}_{10}$	BW ${}_{12}$
Purebred					
BB	397.12 ± 6.1	652.44 ± 7.8	945.89 ± 9.6	1248.78 ± 11.0	1537.73 ± 12.2
CC	402.10 ± 6.0	669.69 ± 7.6	962.53 ± 9.4	1266.64 ± 10.7	1562.90 ± 11.9
RR	363.56 ± 8.6	613.40 ± 10.8	926.14 ± 13.6	1233.30 ± 15.3	1542.73 ± 17.1
Crossbred ${}^{a}$					
BC	442.54 ± 11.5	724.74 ± 14.2	1021.00 ± 17.4	1328.09 ± 19.5	1616.4 ± 21.7
BR	419.37 ± 12.5	693.12 ± 15.3	983.45 ± 18.5	1280.79 ± 20.7	1576.18 ± 23.1
CR	436.69 ± 12.1	718.60 ± 15.6	1014.03 ± 19.4	1323.59 ± 21.8	1635.50 ± 24.4
CB	411.24 ± 12.5	686.06 ± 16.4	969.15 ± 19.9	1262.40 ± 22.1	1562.90 ± 24.7
RB	401.27 ± 11.8	683.48 ± 15.3	975.47 ± 18.7	1281.10 ± 21.2	1583.06 ± 23.8
RC	425.12 ± 11.9	702.82 ± 10.8	1004.53 ± 19.2	1305.12 ± 21.7	1524.73 ± 17.0

${}^{a}$ Sire breed was denoted first. ${}^{***}$ Significant ( p≤0.001 ). ${}^{b}$ BW ${}_{4}$ , BW ${}_{6}$ , BW ${}_{8}$ , BW ${}_{10}$ , and BW ${}_{12}$ are the body weight (g) at 4, 6, 8, 10, and 12 weeks, respectively. BB is Bauscat, CC is Californian, and RR is Baladi Red.

## 4 Crossbreeding effects

### 4.1 Specific heterosis ( $H^{I}$ )

Heterotic effects on body weight shown in Table 4 were significant ( P≤0.001 ). The quantity of heterosis indicates the contemporary relationship between the cross progeny's performance through their parental breeds. A significant positive estimate of $H^{I}$ was ascertained for the growth performance of those crossbred progenies. The influence of $H^{I}$ is formed by the genetic disparity or distinctiveness and genetic distance of the mated groups involved, as well as their ability to complement each other (Abdel-Ghany et al., 2007; Abo-Khadiga et al., 2008; Sanad et al., 2023; Sungkhapreecha et al., 2022; Meky and Altahawy, 2023). Positive and highly significant heterotic effects on body weight are validated by Hagan and Mensah (2019) in a crossbreeding experiment involving New Zealand white (NZW), Blue Vienna (BV), and Chinchilla (CH) breeds. This may have been the result of the existence of non-additive genetic effects between inter-breeds.

**Table 4:** F ratios of least-squares analysis of variance for different factors affecting rabbits' post-weaning body weight traits at different evaluated ages (4–12 weeks of age).

Sources of variation	Df	BW ${}_{4}$ ${}^{a}$	BW ${}_{6}$	BW ${}_{8}$	BW ${}_{10}$	BW ${}_{12}$
Overall heterosis ( $H^{O}$ )	1	39.27 ${}^{***}$	61.67 ${}^{***}$	31.50 ${}^{***}$	22.53 ${}^{***}$	25.72 ${}^{***}$
Purebred differences	2	12.55 ${}^{***}$	18.19 ${}^{***}$	5.79 ${}^{**}$	4.27 ${}^{**}$	5.34 ${}^{**}$
General combining ability	2	1.03	0.56	0.24	0.22	0.92
Maternal ability ( $G^{M}$ )	2	2.73	1.53	1.67	1.61	0.64
Reciprocal effect (SL)	1	0.91	1.76	1.66	3.33	2.56
Specific heterosis ( $H^{I}$ )						
BC	1	9.50 ${}^{**}$	15.19 ${}^{***}$	8.69 ${}^{**}$	5.87 ${}^{*}$	5.27 ${}^{*}$
BR	1	12.42 ${}^{***}$	25.49 ${}^{***}$	10.50 ${}^{***}$	7.04 ${}^{**}$	8.33 ${}^{**}$
CR	1	31.94 ${}^{***}$	40.13 ${}^{***}$	22.43 ${}^{***}$	17.28 ${}^{***}$	21.94 ${}^{***}$

${}^{*}$ Significant ( p≤0.05 ). ${}^{**}$ Significant ( p≤0.01 ). ${}^{***}$ Significant ( p≤0.001 ). ${}^{a}$ BW ${}_{4}$ , BW ${}_{6}$ , BW ${}_{8}$ , BW ${}_{10}$ , and BW ${}_{12}$ are the body weight (g) at 4, 6, 8, 10, and 12 weeks, respectively. BB is Bauscat, CC is Californian, and RR is Baladi Red.

On the contrary, Abdel-Azeem et al. (2007), Eman-Manaa et al. (2011), and Cedano-Castro et al. (2023) described a negative percent heterosis for post-weaning growth traits in the different breeds of rabbits. The least-squares mean of different breed groups (coupled with those estimates of heterosis) indicated that the cross (CR) significantly ( P≤0.01 or P≤0.001 ) surpassed those of other crossbreds in body weights at all ages studied, followed by the cross (BR). The poorest figures were attained by BC (Table 5). El-Bayomi et al. (2012), Abdel-Hamid (2015), and Meky and Altahawy (2023) showed strong individual heterosis for New Zealand × Californian crossbreds on body weights at all ages studied. Eman-Manaa et al. (2011) did not perceive any significant heterosis for BW at all ages studied except at 4 weeks of age, where results showed positive heterosis for the cross of Baladi Black 
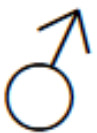
 × New Zealand 
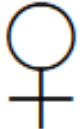
.

**Table 5:** Constant ± standard error for crossbreeding effects of rabbits' post-weaning body weight traits at different evaluated ages (4–12 weeks of age).

Crossbreeding effect	BW ${}_{4}$	BW ${}_{6}$	BW ${}_{8}$	BW ${}_{10}$	BW ${}_{12}$
Overall heterosis ( $H^{O}$ )	210.68 ± 33.6 ${}^{***}$	337.77 ± 43.0 ${}^{***}$	298.53 ± 53.2 ${}^{***}$	283.06 ± 59.6 ${}^{***}$	337.91 ± 66.6 ${}^{***}$
Purebreds					
BB	-21.80 ± 12.5 ${}^{*}$	-43.31 ± 9.7 ${}^{***}$	-3.10 ± 15.6	2.67 ± 17.6	11.44 ± 19.6
CC	-73.53 ± 12.5 ${}^{***}$	-75.19 ± 10.0 ${}^{***}$	-53.03 ± 15.6 ${}^{***}$	-50.89 ± 17.6 ${}^{**}$	-61.88 ± 19.6 ${}^{*}$
RR	95.33 ± 18.3 ${}^{***}$	118.50 ± 10.6 ${}^{***}$	56.14 ± 23.1 ${}^{*}$	48.21 ± 25.9	50.45 ± 28.9
General combining ability (GCA)					
BB	0.08 ± 21.4	-3.54 ± 27.4	-14.10 ± 33.8	-19.82 ± 37.9	-55.99 ± 42.3
CC	27.30 ± 21.9	28.04 ± 28.8	24.26 ± 35.0	24.10 ± 39.1	39.16 ± 43.8
RR	-27.39 ± 21.9	-24.50 ± 28.3	-10.16 ± 34.8	-4.28 ± 39.0	16.83 ± 43.5
Maternal ability ( $G^{M}$ )					
BB	-49.40 ± 21.3 ${}^{*}$	-48.34 ± 27.7	-59.83 ± 33.5	-65.38 ± 37.4	-46.66 ± 41.8
CC	19.72 ± 21.9	22.91 ± 28.2	42.35 ± 34.9	47.21 ± 39.0	31.14 ± 43.6
RR	29.68 ± 21.5	25.43 ± 27.9	17.49 ± 34.4	18.17 ± 38.7	15.52 ± 43.2
Reciprocal effect (sex-linked; SL)					
BC	31.29 ± 15.3 ${}^{*}$	38.69 ± 19.7 ${}^{*}$	51.85 ± 23.9 ${}^{*}$	65.69 ± 26.6 ${}^{**}$	53.54 ± 29.8
CB	-31.29 ± 15.3 ${}^{*}$	-38.69 ± 19.7 ${}^{*}$	-51.85 ± 23.9 ${}^{*}$	-65.69 ± 26.6 ${}^{**}$	-53.54 ± 29.8
BR	18.10 ± 14.9	9.65 ± 19.4	7.98 ± 23.5	-0.31 ± 26.3	-6.88 ± 29.5
RB	-18.10 ± 14.9	-9.65 ± 19.4	-7.98 ± 23.5	+0.31±26.3	+6.88±29.5
CR	11.57 ± 15.4	15.78 ± 19.9	9.51 ± 24.9	18.48 ± 28.0	29.39 ± 31.3
RC	-11.57 ± 15.4	-15.78 ± 19.9	-9.51 ± 24.9	-18.48 ± 28.0	-29.39 ± 31.3
Specific heterosis ( $H^{I}$ )					
BC	54.56 ± 17.7 ${}^{**}$	88.67 ± 22.8 ${}^{***}$	81.74 ± 27.7 ${}^{**}$	75.07 ± 31.0 ${}^{*}$	79.43 ± 34.6 ${}^{*}$
BR	59.96 ± 17.0 ${}^{***}$	110.77 ± 21.9 ${}^{***}$	86.90 ± 26.8 ${}^{***}$	79.51 ± 30.0 ${}^{**}$	96.78 ± 33.5 ${}^{**}$
CR	96.16 ± 17.0 ${}^{***}$	138.33 ± 21.8 ${}^{***}$	129.89 ± 27.4 ${}^{***}$	128.47 ± 30.9 ${}^{***}$	161.70 ± 34.5 ${}^{***}$

${}^{+}$ Traits are as in Table 2. BB is Bauscat, CC is Californian, and RR is Baladi Red. ${}^{*}$ Significant ( p≤0.05 ). ${}^{**}$ Significant ( p≤0.01 ). ${}^{***}$ Significant ( p≤0.001 ).

### 4.2 Purebred difference (linear functions and standard error)

Results of linear contrasts between BB, CC, and RR rabbits for body weight (g) are presented in Table 4 (ANOVA) and Table 5 (linear functions). Differences due to purebred differences were significant ( P≤0.01 or P≤0.001 ) at all ages studied (Table 4). In this respect, Khalil et al. (2002), Abdel-Ghany et al. (2007), Nwakpu et al. (2015), and Palka et al. (2023) showed significant purebred differences. Effect estimates declared a general superiority of RR on BB and CC rabbits in most ages under consideration but insignificantly in later stages of life starting from the 10th week of age.

However, this superiority of Baladi Red rabbits could be due to their adaptability to environmental conditions in Egypt compared to the other two exotic breeds. This can be attributed to the genetic compositions that enable them to adapt to the local ecological conditions (Ragab et al., 2022). It should be mentioned that a decrease in performance always accompanies the acclimatization process. However, for a given pure breed, being superior does not guarantee that it will result in superior crossings when used in a crossbreeding program.

### 4.3 General combining ability (GCA)

Procedures to identify superior cross-combinations are important in all aspects of animal breeding. The performance of a breed or strain in hybrids (cross-combination) may be evaluated in terms of general combined abilities, maternal ability, and reciprocal effect.

The analysis of variance for the effect of GCA on all studied body weights was non-significant ( P>0.05 ) among the breeds (BB, CC, and RR; Table 4). Though insignificant, BB and RR rabbits had a negative value of GCA. In contrast, CC rabbits had a positive and superior value of general combining ability for most studied body weights (Table 5).

These results agreed, as reported by Eman-Manaa et al. (2011), El-Bayomi et al. (2012), and Kariman-Farg et al. (2021). Conversely, Adenaike et al. (2013), Egena et al. (2012), Kabir et al. (2014) and Setiaji et al. (2022) confirmed highly significant ( P≤0.01 ) differences in BW at different ages for different breeds due to GCA. The non-significant effect of GCA is because these traits are affected by non-additive gene effects (i.e., dominance, over-dominance, and epistasis). Thus, crossing is the chosen procedure for improving such traits. Therefore, improving the nicking ability between such breeds (with non-significant GCA effects) and exploiting recurrent and reciprocal selection would be a solution.

### 4.4 Maternal ability ( $G^{M})$

Maternal ability could be appraised as a peculiarity of a given breed of rabbits in any maternal line of the crossbreeding plan (Abo-Khadiga et al., 2008; Mínguez et al., 2012, 2015). However, maternal abilities ( $G^{M}$ ) had insignificant effects on all BW traits (Table 4). Comparable results were obtained by Khalil and Afifi (2000), who showed non-significant ( P>0.05 ) $G^{M}$ effects on most BW. Conversely, Khalil et al. (2002) displayed significant effects ( P≤0.01 ) of $G^{M}$ on the BW of different breeds of rabbits. Regarding linear constants (Table 5), it was found that, apart from significance, the apparent highest estimates at the initial stages of life were for RR (at 4 and 6 weeks of age), while later, the highest estimate was for CC (8–12 weeks of age). However, BB showed the poorest mothering ability compared to both CC and RR breeds.

### 4.5 Reciprocal or sex-linked (SL) effects

Sex-linked effects express the additive effect of genes carried on the sex chromosome. Because of that, non-homologous sex contributes unequally to its progeny, and the use of a breed as a sire line has a consequence that differs from that when it is used as a dam line. Body weights of rabbits at all studied ages were non-significantly ( P>0.05 ) affected by SL (reciprocal) effects (see Table 4). Consequently, using a breed as a sire or dam line would not accumulate any further advantage for improvement. Similarly, Eman-Manaa et al. (2011), El-Bayomi et al. (2012), Adenaike et al. (2013), Kabir et al. (2014), Kariman-Farg et al. (2021) and Abdullah (2022) reported non-significant ( P>0.05 ) differences in body weights due to sex-linked effects.

## 5 Conclusion

The results showed that hybrid vigor is promising in the evaluated crossbreeding plan considering post-weaning body weight performance until 12 weeks of age. However, crossing Californian with Baladi Red (CR) and Bauscat with Baladi Red (BR) specifically would be better, surpassing the other breed combinations (BCs) in producing broiler rabbits under Egyptian conditions due to that heterotic effect. The development of these hybrid assemblies is promising for distribution on an industrial scale under Egyptian conditions, benefiting the resultant perceived heterotic effects.

## Data Availability

The datasets used and analyzed during the current study are available from the corresponding author upon reasonable request.
